# Na^+^/K^+^-ATPase Inhibition Partially Mimics the Ethanol-Induced Increase of the Golgi Cell-Dependent Component of the Tonic GABAergic Current in Rat Cerebellar Granule Cells

**DOI:** 10.1371/journal.pone.0055673

**Published:** 2013-01-31

**Authors:** Marvin R. Diaz, Aya Wadleigh, Shyam Kumar, Erik De Schutter, C. Fernando Valenzuela

**Affiliations:** 1 Department of Neurosciences, University of New Mexico Health Sciences Center, Albuquerque, New Mexico, United States of America; 2 Computational Neuroscience Unit, Okinawa Institute of Science and Technology, Okinawa, Japan; 3 Department of Theoretical Neurobiology, University of Antwerp, Wilrijk, Belgium; University of North Dakota, United States of America

## Abstract

Cerebellar granule cells (CGNs) are one of many neurons that express phasic and tonic GABAergic conductances. Although it is well established that Golgi cells (GoCs) mediate phasic GABAergic currents in CGNs, their role in mediating tonic currents in CGNs (CGN-I_tonic_) is controversial. Earlier studies suggested that GoCs mediate a component of CGN-I_tonic_ that is present only in preparations from immature rodents. However, more recent studies have detected a GoC-dependent component of CGN-I_tonic_ in preparations of mature rodents. In addition, acute exposure to ethanol was shown to potentiate the GoC component of CGN-I_tonic_ and to induce a parallel increase in spontaneous inhibitory postsynaptic current frequency at CGNs. Here, we tested the hypothesis that these effects of ethanol on GABAergic transmission in CGNs are mediated by inhibition of the Na^+^/K^+^-ATPase. We used whole-cell patch-clamp electrophysiology techniques in cerebellar slices of male rats (postnatal day 23–30). Under these conditions, we reliably detected a GoC-dependent component of CGN-I_tonic_ that could be blocked with tetrodotoxin. Further analysis revealed a positive correlation between basal sIPSC frequency and the magnitude of the GoC-dependent component of CGN-I_tonic_. Inhibition of the Na^+^/K^+^-ATPase with a submaximal concentration of ouabain partially mimicked the ethanol-induced potentiation of both phasic and tonic GABAergic currents in CGNs. Modeling studies suggest that selective inhibition of the Na^+^/K^+^-ATPase in GoCs can, in part, explain these effects of ethanol. These findings establish a novel mechanism of action of ethanol on GABAergic transmission in the central nervous system.

## Introduction

GABA – the main inhibitory neurotransmitter in the mammalian brain – acts via activation of receptors located at synaptic and extrasynaptic sites. Extrasynaptic GABA_A_ receptors (GABA_A_Rs) with unique subunit compositions have been characterized in different brain regions. In the CA1 and CA3 hippocampal subfields [1] and cortical layer 5 [2], receptors composed of α5βγ subunits have been identified. Receptors containing α4βδ subunits are expressed in the dentate gyrus, thalamus, striatum, and neocortex [3], [4], while receptors containing α6βδ subunits are exclusively expressed in cerebellar granule neurons (CGNs) [3], [4]. Extrasynaptic GABA_A_Rs are activated by ambient levels of GABA that can be in the tens of nanomolar to micromolar range [5]. The high affinity for GABA of extrasynaptic GABA_A_Rs endows them with the ability to sense relatively low concentrations of this transmitter [6]. Moreover, although ambient GABA levels can produce significant desensitization of extrasynaptic GABA_A_Rs, an appreciable residual level of receptor activity persists under these conditions, generating a tonic current that significantly dampens neuronal excitability [7], [8]. In the case of CGNs, synapses are ensheathed by a glomerulus that is thought to decrease GABA diffusion [9], [10]. A recent study suggests that astrocytes can release GABA via the Ca^2+^-activated anion channel, bestrophin 1, and that this process is responsible for generating ∼50–70% of the tonic GABAergic current in CGNs (CGN-I_tonic_) [11], [12], but some of the findings of this report are controversial [13]. The sources of GABA responsible for the remaining 30–50% of the CGN-I_tonic_ have not been thoroughly characterized. Initial CGN slice electrophysiological recordings suggested that accumulation of GABA released in an action potential-dependent manner from cerebellar Golgi cells (GoCs) significantly contributes to the GABA pool that activates extrasynaptic receptors in young (postnatal day (P) 7–20), but not older (P35–53) rats [8], [9], [10], [14], [15]. Based on these studies, it was concluded that spontaneous action potential-dependent GABA release does not play a major role in CGN-I_tonic_ generation in older rats [16], [17].

More recent studies have provided evidence challenging this prevailing view. Slice recordings from our laboratory demonstrated that GABA release driven by spontaneous firing of GoCs contributes to the generation of CGN-I_tonic_ in P30–45 male rats at 31°C [18]. Application of the antagonist of voltage-gated Na^+^ channels, tetrodotoxin (TTX), significantly decreased CGN-I_tonic_ by approximately 25% in slices from these animals. This effect was associated with a large decrease (∼75%) in the frequency of spontaneous inhibitory postsynaptic currents (sIPSCs) in CGNs. In agreement with these findings, an even more robust decrease in both CGN-I_tonic_ (∼50%) and sIPSC frequency (∼97%) was observed at 37–38°C using cerebellar slices from adult (P68±3) male mice [7]. These results strongly suggest that spontaneous action potential-dependent GABA release from GoCs plays a more central role in CGN-I_tonic_ generation than previously thought. It is therefore important to better characterize the regulation of this CGN-I_tonic_ component under physiological and pathophysiological conditions.

Studies suggest that ethanol (EtOH) is a positive modulator of the GoC-dependent component of CGN-I_tonic_ [18], [19]. Specifically, acute EtOH exposure increases both the frequency of sIPSCs driven by GoC firing and the magnitude of CGN-I_tonic_, and these effects are blocked by TTX [18]. Recordings from GoCs revealed that EtOH dose-dependently increases spontaneous GoC firing, an effect that appears to be, at least in part, a consequence of slight inhibition of the Na^+^/K^+^-ATPase [18], [19], [20]. *In vivo* electrophysiological studies indicate that acute EtOH exposure both increases spontaneous GoC firing and inhibits sensory responses of CGNs [21], [22]. Therefore, the EtOH-induced increase of CGN-I_tonic_ could be one of the underlying mechanisms responsible for the motor coordination alterations associated with acute intoxication.

In this study, we further examined the contribution of spontaneous GABA release from GoCs to CGN-I_tonic_ and its modulation by acute EtOH exposure. Using slice electrophysiological techniques, we found evidence for a direct relationship between sIPSC frequency and the GoC-dependent component of the tonic current. Moreover, slight inhibition of the Na^+^/K^+^-ATPase with a submaximal concentration of ouabain partially mimicked the effects of acute EtOH exposure on both sIPSC frequency and the GoC-dependent component of CGN-I_tonic_. Modeling studies suggest that selective inhibition of the Na^+^/K^+^-ATPase in GoCs can, in part, account for the effects of EtOH on GABAergic transmission at CGNs.

## Methods

### Animals

Male Sprague Dawley rats (P23–30) from Harlan (Indianapolis, IN) were used for this study. Animals were group-housed and received food and water *ad libitum*. All animal procedures were approved by the UNM-Health Sciences Center Institutional Animal Care and Use Committee and conformed to National Institutes of Health Guidelines.

### Brain Slice Preparation

Animals were sacrificed by rapid decapitation under deep anesthesia with ketamine (250 mg/kg i.p.). Brains were rapidly removed and submerged for 2 minutes in an ice-cold solution containing (in mM): 220 Sucrose, 2 KCl, 1.25 NaH_2_PO_4_, 26 NaHCO_3_, 12 MgSO_4_, 10 Glucose, 0.2 CaCl_2_ and 0.43 ketamine that was pre-equilibrated with 95% O_2_/5% CO_2_. The vermis of the cerebellum was sliced in this solution at 200 µm using a vibrating slicer (Leica Microsystems, Bannockburn, IL). Slices were then transferred to a chamber containing artificial cerebrospinal fluid (aCSF) and allowed to recover for 40 minutes at 35–36°C. The aCSF contained (in mM): 126 NaCl, 2 KCl, 1.25 NaH_2_PO_4_, 26 NaHCO_3_, 10 Glucose, 1 MgSO_4_, 2 CaCl_2_ and 0.4 ascorbic acid and was continuously bubbled with 95% O_2_/5% CO_2_.

### Whole Cell Patch-Clamp Electrophysiology

The whole-cell patch-clamp configuration was used to record tonic and phasic GABA_A_R-mediated currents. Recordings were performed in a chamber perfused with aCSF at a rate of 2–3 ml/min and maintained at 32–33°C. Neurons were visualized using infrared-differential interference contrast microscopy and recordings were performed with Axopatch 200B or MultiClamp 700B amplifiers (Molecular Devices, Sunnyvale, CA). CGNs were identified on the basis of their location in the CGN layer, morphology (small and round), and capacitance (∼2–5 pF). Patch pipettes (tip resistance = 3–5 MΩ) were filled with an internal solution containing (in mM): 135 KCl, 10 HEPES, 2 MgCl_2_, 0.5 EGTA, 5 Mg-ATP, 1 Na-GTP, and 1 QX314-(Br), pH 7.25, osmolarity 280 to 290 mOsm. The holding potential was −70 mV. Recordings in which the access resistance did not change >20% throughout the duration of the experiment were included for analysis. GABAergic synaptic transmission was isolated by blocking AMPA and NMDA receptors using kynurenic acid (1 mM) and D,L-APV (50 µM), respectively. During application of glutamate antagonists, neurons were allowed to equilibrate (∼5 min) prior to beginning the experiment. Data were acquired in gap-free mode at 10 kHz and filtered at 2 kHz.

Data were analyzed with Clampfit-10 (Molecular Devices) or Minianalysis (Synaptosoft, Decatur, GA). As previously shown, the CGN-I_tonic_ amplitude was calculated by fitting a Gaussian distribution to an all-point histogram for every minute of the recording, constraining the fit to eliminate a contribution from sIPSCs [13]. CGN-I_tonic_ amplitude was defined as the mean current recorded under control conditions minus that recorded in the presence of gabazine (10 µM). Based on our previous study, ouabain (0.1 µM) was applied for 5 min followed by an additional 5 min to allow stabilization of the action of this long-lasting Na^+^/K^+^-ATPase blocker; under these conditions, we previously showed that ouabain mimics the effect of acute exposure to 40 mM EtOH on GoC firing [19].

### Statistics

Data were statistically analyzed with Prizm 5 (Graphpad, San Diego, CA) and MiniAnalysis (Synaptosoft, Decatur, GA). Data were initially analyzed with the D'Agostino-Pearson omnibus normality test. If data were normally distributed, they were analyzed using parametric tests. If this was not the case, then non-parametric tests were used. Data are presented as mean ± SEM and a *p*≤0.05 was considered to be statistically significant.

### Drugs and Chemicals

D,L-APV, gabazine hydrobromide, and QX-314 (Br^−^) were purchased from Tocris (Ellisville, MO). TTX was from Calbiochem (San Diego, CA). All other drugs and chemicals were from Sigma-Aldrich (St. Louis, MO).

### Computer Modeling

A model, based on a granular layer network model of the cerebellum [23], was built using the NEURON (version 7.1) simulation environment [24] with a spatial dimension of 0.15×1.5 mm and three 2D populations. Mossy fibers, CGNs (soma and parallel fibers) and GoCs are represented. GoCs were based on a previously published model [25]. Na^+^/K^+^-ATPase was simulated according to the equations given in table S10 of Takeuchi et al., 2006 [26]. The effect of ouabain was simulated by systematically reducing the Na^+^/K^+^-ATPase component [19]. CGNs were approximated as a single compartment model, derived from a previously published multi-compartmental model [27]. The connectivity between neurons in the network was based on published anatomical connectivity patterns of the CGN layer [28]. The network also included gap junctions between GoCs. CGN IPSCs evoked by GoC stimulation were characterized by a rapid rise phase followed by a biphasic decay phase [8]. The GoC-to-CGN IPSCs in the model had two components: a phasic component with rapid rise (0.31 ms) and decay time (8.8 ms) and a spillover component with slow rise (6.8 ms) and decay time (232.5 ms). Both components were modeled as probabilistic synaptic transmission with a probability of release of 0.35. The amplitude of the spillover component was set to 15% of the phasic component according to experimental results [7]. CGN IPSPs were detected systematically using MATLAB® computer program (MathWorks, Natick, MA). A total of 350–650 IPSPs recorded in 50 CGNs during a 1 second run of the model were analyzed for each condition. The amplitude of CGN IPSPs was measured relative to the preceding minimum; only events that were temporally separated by more than 22 ms were analyzed to eliminate temporal summation. Statistical analysis of data was performed in MATLAB using a two-tailed unpaired t-test. Data are presented as mean ± SEM and a *p* value<0.01 was considered to be statistically significant.

## Results

### Characterization of the relationship between CGN-I_tonic_ and sIPSCs

**Figure 1:**
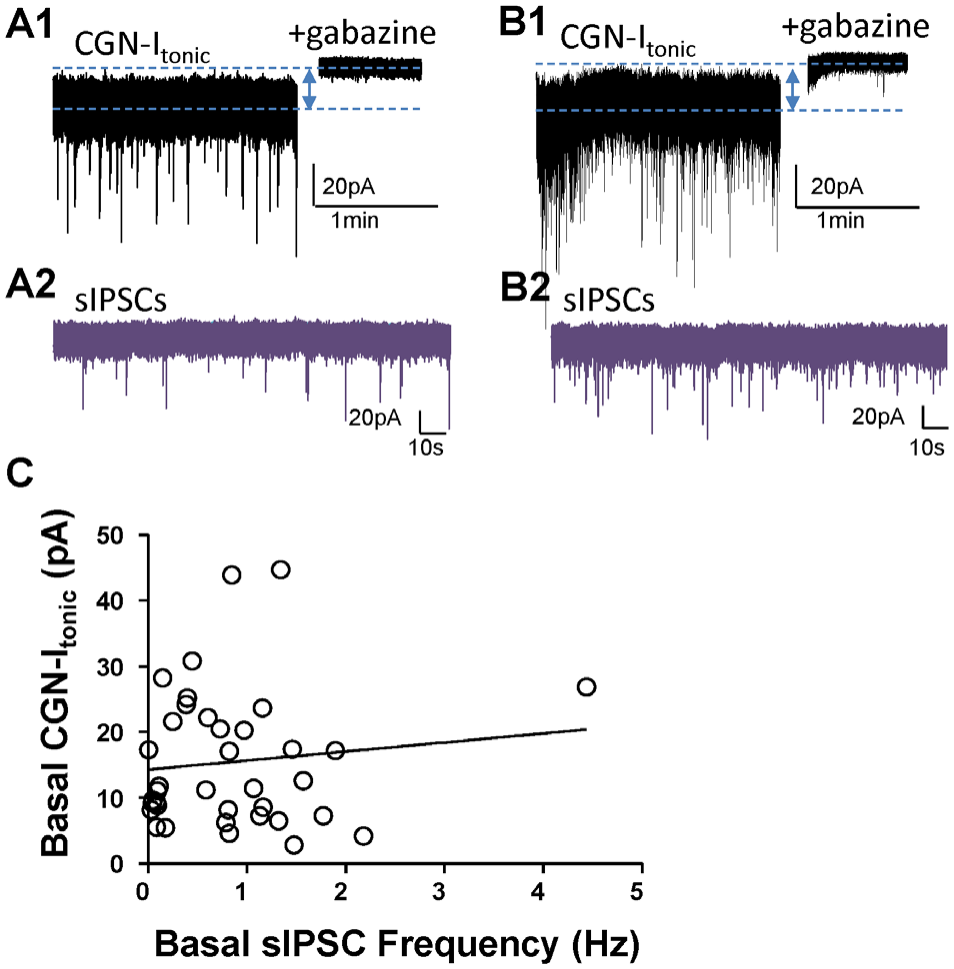
The magnitude of tonic current amplitudes and sIPSC frequencies are not correlated. Exemplar traces of two cells with similar tonic current amplitudes (A1 – 21.6 pA and B1 – 20.5 pA), but different sIPSC frequencies (A2 – 0.24 Hz and B2 – 0.73 Hz). Dashed lines and arrows illustrate that the CGN-I_tonic_ amplitude during baseline and in the presence of gabazine is nearly identical in both exemplar traces. (C) Plot of basal sIPSC frequency (Hz) versus basal current amplitude (pA) shows no correlation (slope not significantly different from zero, *p*>0.05, r^2^ = 0.012).

To characterize the relationship between phasic and tonic GABAergic transmission, we quantified sIPSC frequency as a function of CGN-I_tonic_ amplitude. We found no significant correlation between basal CGN-I_tonic_ amplitude and sIPSC frequency. Fig. 1A–B shows sample recordings from two CGNs with similar I_tonic_ amplitudes and substantially different sIPSC frequencies. Fig. 1C illustrates the lack of correlation between sIPSC frequency and CGN-I_tonic_ amplitude (Fig. 1C; slope = 1.37, 95% confidence intervals = [−2.75, 5.50], *p*>0.05, r^2^ = 0.012).

### GoC excitability significantly contributes to tonic GABAergic currents and sIPSCs

**Figure 2:**
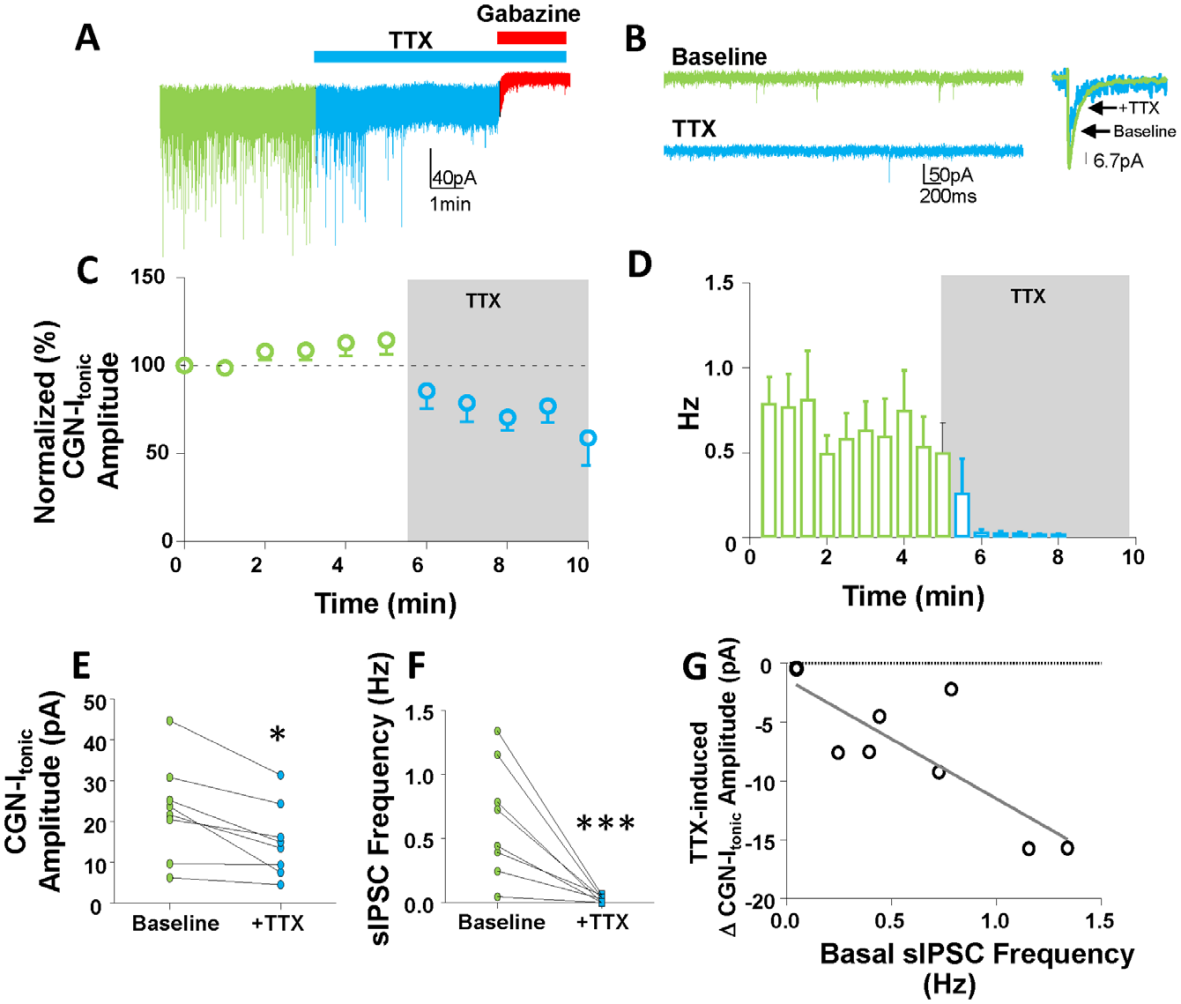
TTX-induced decreases of CGN-I_tonic_ and sIPSCs are correlated. Representative traces of the effect of TTX and subsequent gabazine application on (A) CGN-I_tonic_ and (B) sIPSC frequency and amplitude. Time courses of the effect of TTX on (C) normalized amplitude of the CGN-I_tonic_ and (D) sIPSC frequency. Plots of the (E) CGN-I_tonic_ and (F) sIPSC frequencies of individual cells before and after TTX; * *p*<0.05, paired t-test. (G) Correlation between basal sIPSC frequency (Hz) and TTX-induced change in tonic current amplitude (pA); slope significantly different from zero, *p*<0.05, r^2^ = 0.63.

Given the current hypothesis that action potential-dependent GABA release from GoCs significantly contributes to the pool of GABA that activates extrasynaptic GABA_A_Rs, we investigated the impact of blocking spontaneous GoC firing on CGN-I_tonic_. We found that TTX significantly reduces CGN-I_tonic_ amplitude by 32.36±6.3% (Fig. 2A, C and E; baseline = 23.05±4.1 pA, TTX = 15.20±2.9 pA, *p*<0.01, paired t-test; n = 8). TTX almost completely eliminated all sIPSCs, reducing their frequency by 94.76±2.11% (Fig. 2A, B, D and F, baseline = 0.64±0.16 Hz, TTX = 0.03±0.01 Hz, *p*<0.01, paired t-test; n = 8). TTX also significantly decreased the amplitude of sIPSCs by 50.54±15.65% (baseline = 53.51±4.97 pA, TTX = 26.45±8.54 pA, *p*<0.05, paired t-test; n = 8; see sample average traces in Fig. 2B). We investigated whether the magnitude of the TTX effect on CGN-I_tonic_ was related to the basal sIPSC frequency. We found that the basal sIPSC frequency was significantly correlated with the TTX-induced change in tonic current amplitude (Fig. 2G, r^2^ = 0.63, slope = −10.16, 95% confidence intervals = [−17.81,−2.50], *p*<0.05 from zero; n = 8).

### Effect of ouabain and EtOH on CGN-I_tonic_

**Figure 3:**
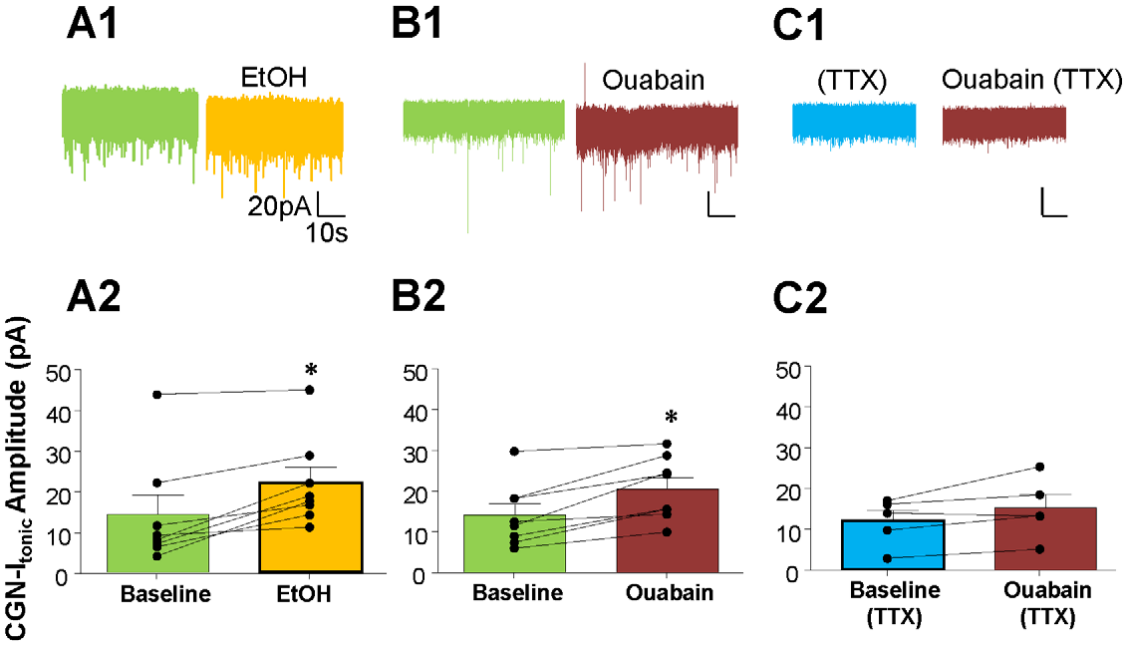
Ouabain partially mimics the EtOH-induced, action potential-dependent potentiation of CGN-I_tonic_. Exemplar traces of the effect of (A1) 80 mM EtOH, (B1), 0.1 µM ouabain, and (C1) 0.1 µM ouabain in the presence of TTX on the CGN-I_tonic_. Summary of the effect of (A2) EtOH, (B2) ouabain, and (C2) TTX+ouabain on the amplitude of CGN-I_tonic_. * *p*<0.05, paired t-test.

We previously demonstrated that slight inhibition of the Na^+^/K^+^-ATPase with a low concentration of ouabain increases GoC firing in a similar fashion to acute EtOH exposure [19]. Here, we tested whether the ouabain-induced increase in GoC firing potentiates CGN-I_tonic_ and if this effect is similar to that of acute EtOH exposure [18]. We measured the change in CGN-I_tonic_ in the presence of 80 mM EtOH (Fig. 3A1) or 0.1 µM ouabain (see Methods and [19]) (Fig. 3B1). We found that EtOH significantly increases CGN-I_tonic_ amplitude (Fig. 3A2, baseline = 14.21±4.66 pA, EtOH = 21.93±3.79 pA, *p*<0.05, paired t-test; n = 8), an effect that was at least partially reversible in 6 out of 8 cells after a 5 min washout (average = 19.26±2.82 pA). Ouabain (0.1 µM) also significantly increased CGN-I_tonic_ amplitude (Fig. 3B2, baseline = 14.05±2.76 pA, ouabain = 20.52±2.72 pA at t = 10 min, *p*<0.05, paired t-test; n = 8). In agreement with previous results [19], the effect of ouabain was not reversible, consistent with its high affinity for the Na^+^/K^+^-ATPase.

The effect of EtOH on CGN-I_tonic_ was previously shown to be blocked by TTX [18]. Therefore, we tested the effect of ouabain on the CGN-I_tonic_ in the presence of TTX (Fig. 3C1). In the presence of TTX, ouabain did not significantly alter CGN-I_tonic_ amplitude (Fig. 3C2; TTX = 11.93±2.59pA, TTX+ouabain = 15.09±3.33 pA at t = 10 min, *p*>0.05, paired t-test; n = 5).

### Effect of ouabain and EtOH on sIPSCs in CGNs

**Figure 4:**
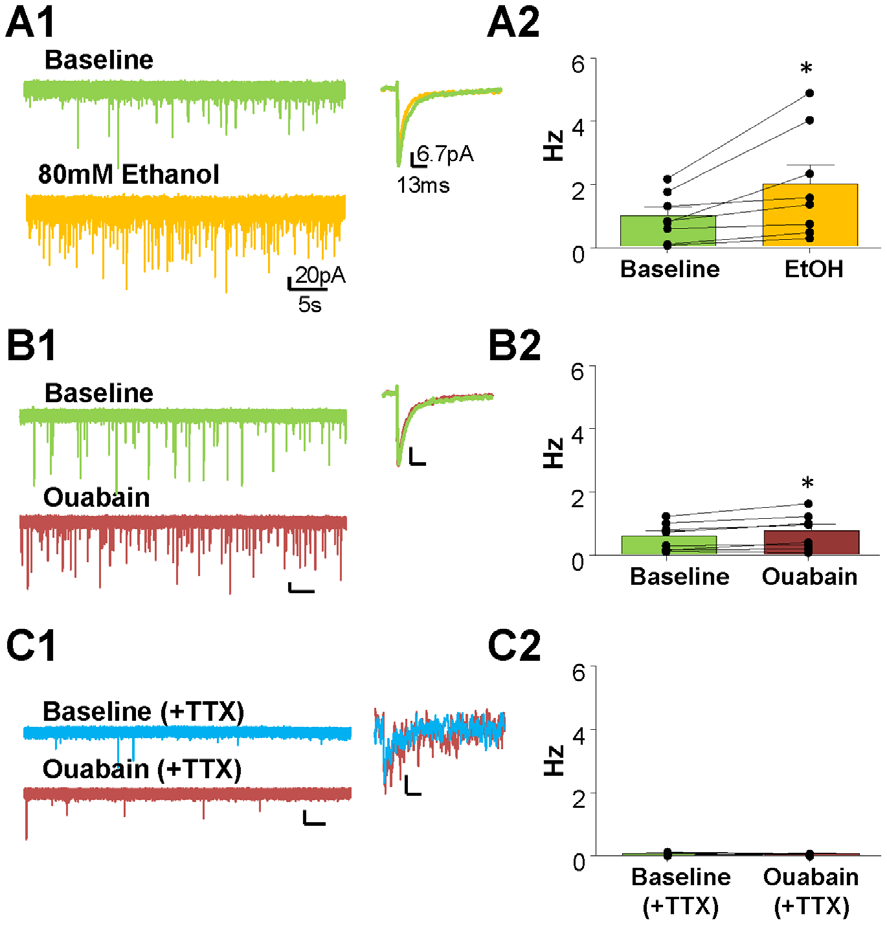
Ouabain only partially mimics the EtOH-induced facilitation of sIPSCs. Representative traces of the effects of (A1) 80 mM EtOH, (B1) 0.1 µM ouabain, and (C1) TTX+ouabain on sIPSC frequency and amplitude (average green traces = baseline; blue traces = baseline (+TTX); yellow = EtOH; red = ouabain). Summary of the effect of (A2) EtOH, (B2) ouabain, and (C2) TTX+ouabain on sIPSC frequency. * *p*<0.05, *** *p*<0.01 paired t-test.

We found that 80 mM EtOH significantly increases sIPSC frequency (Fig. 4A1–A2, baseline = 0.96±0.26 Hz, EtOH = 1.97±0.59 Hz, *p*<0.05, paired t-test; n = 8), but not amplitude (baseline = 53.13±5.45 pA, EtOH = 51.82±4.57 pA, *p*>0.05 paired t-test; n = 8; see trace on the right of Fig. 4A1) or decay time (baseline = 11.13±0.81 ms, EtOH = 10.87±0.66 ms, *p*>0.05 paired t-test; n = 8). Ouabain (0.1 µM) also increased sIPSC frequency (Fig. 4B1–B2, baseline = 0.57±0.15 Hz, ouabain = 0.73±0.19 Hz at 10 min, *p*<0.05, paired t-test; n = 8), but to a lesser extent than EtOH (30.28±17.9% vs 150.1±43.62%, respectively), with no effect on the amplitude (baseline = 53.52±5.63 pA, ouabain = 56.10±6.46 pA, *p*>0.05 paired t-test; see trace on the right of Fig. 4B1).

We also found that in the presence of TTX, there is no significant effect of ouabain on mIPSC frequency (Fig. 4C1–C2, TTX = 0.06±0.02 Hz, ouabain = 0.04±0.013 Hz at 10 min, *p*>0.05, paired t-test; n = 3) with no change in amplitude (mIPSC amplitude – baseline = 31.37±1.28 pA, ouabain = 33.49±3.01 pA, *p*>0.05 paired t-test; n = 3; see trace on the right of Fig. 4C1).

### Relationship between the effect EtOH on CGN-I_tonic_ and sIPSCs

**Figure 5:**
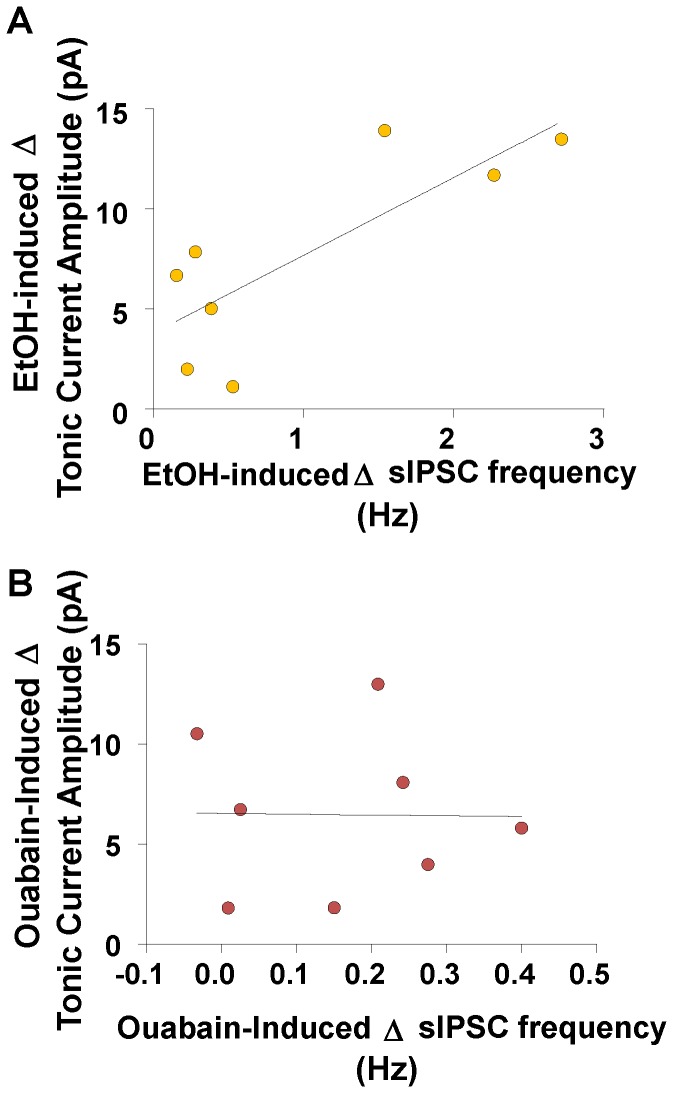
The EtOH- (but not the ouabain-) induced change of tonic current is correlated with the EtOH-induced change in sIPSC frequency. Correlations between the (A) EtOH- or (B) ouabain-induced change in sIPSC frequency (Hz) versus EtOH- or ouabain-induced change in tonic current amplitude (pA); EtOH – slope = 3.88, *p*<0.05 from zero, r^2^ = 0.063; ouabain – slope = −0.38; *p*>0.05, r^2^ = 0.0002.

We assessed the relationship between the EtOH- or ouabain-induced change in sIPSC frequency and CGN-I_tonic_ amplitude. The EtOH-induced change in sIPSC frequency was significantly correlated with the change in CGN-I_tonic_ (Fig. 5A, slope = 3.88, 95% confidence intervals = [0.96, 6.80], *p*<0.05, r^2^ = 0.64). In contrast, the ouabain-induced change in sIPSC frequency was not significantly correlated to the change in CGN-I_tonic_ (Fig. 5B, slope = −0.38, 95% confidence intervals = [−26.86, 26.09], *p*>0.05, r^2^ = 0.0002).

### Computer modeling of effects of EtOH and ouabain on GABAergic transmission at GoC-CGN synapses

**Figure 6:**
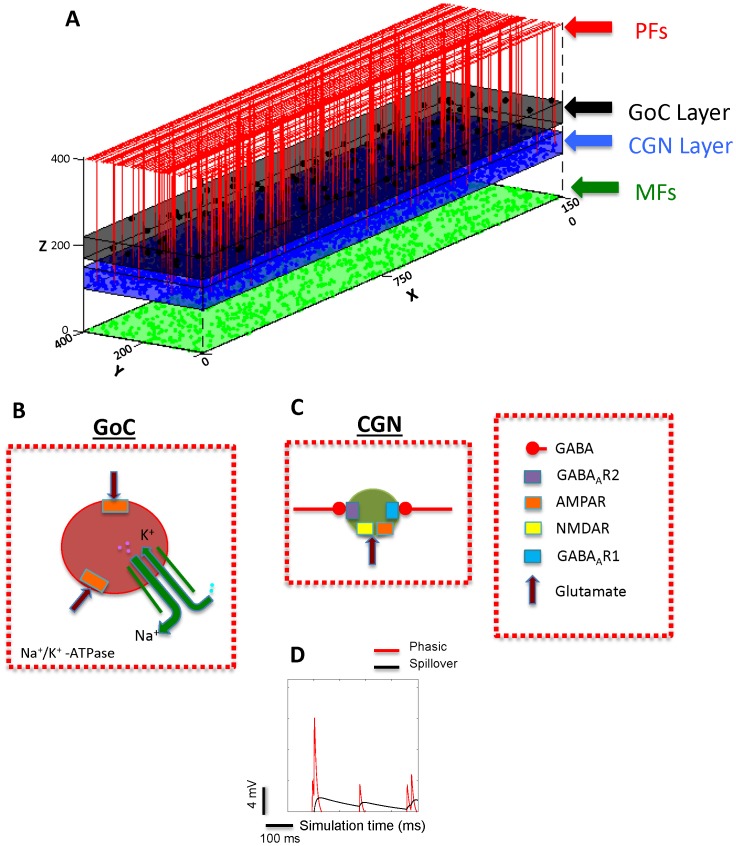
Structure of the network computer model. (A) The model consists of three populations. A two dimensional layer represents each population. The GoC population is indicated by black color, CGN in blue, mossy fibres (MFs) in green, and the parallel fibers (PFs) emanating from CGNs are shown in red. (B) Postsynaptic receptors of a modeled GoC and neurotransmitters activating them. Each modeled GoC has AMPA receptors activated by glutamate either from MFs or PFs. The Na^+^/K^+^-ATPase has been incorporated in the soma of the model. (C) CGNs have NMDA and AMPA receptors activated by glutamate from MFs and GABA receptors activated by GABA neurotransmission from GoCs. CGNs also have two types of GABA_A_ receptors on them: GABA_A_R1 (phasic) that has rapid rise (0.31 ms) and decay time (8.8 ms) and GABA_A_R2 (perisynaptic) that has slow rise (6.8 ms) and decay time (232.5 ms). D) Sample traces illustrating the events mediated by synaptic and perisynaptic receptors.

We investigated the effect of ouabain on the frequency and amplitude of CGN IPSPs by computer modeling (Fig. 6). The concentration of ouabain used in the model was 0.125 µM. All simulations were carried out in the absence of mossy fiber input with a spontaneously active GoC population. CGN IPSPs caused by GoCs have two GABAergic components: a phasic component with rapid rise and decay time and a perisynaptic “spillover” component with slow rise and decay time (Fig. 6D). The inter-event interval and frequency of IPSPs was measured in 50 CGNs.

**Figure 7:**
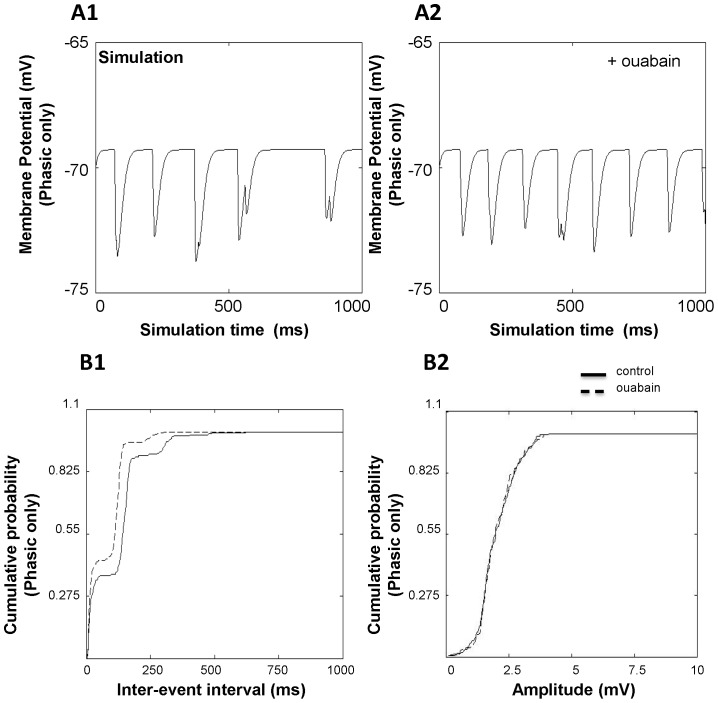
Effect of ouabain on the frequency and amplitude of simulated CGN IPSPs (phasic component only). IPSP traces for (A1) control condition and (A2) in the presence of ouabain with phasic component only. Effect of ouabain on (B1) inter-event interval and (B2) amplitude in the presence of the phasic component only. Traces represent recorded inter-event intervals and amplitudes of 50 CGNs.

**Figure 8:**
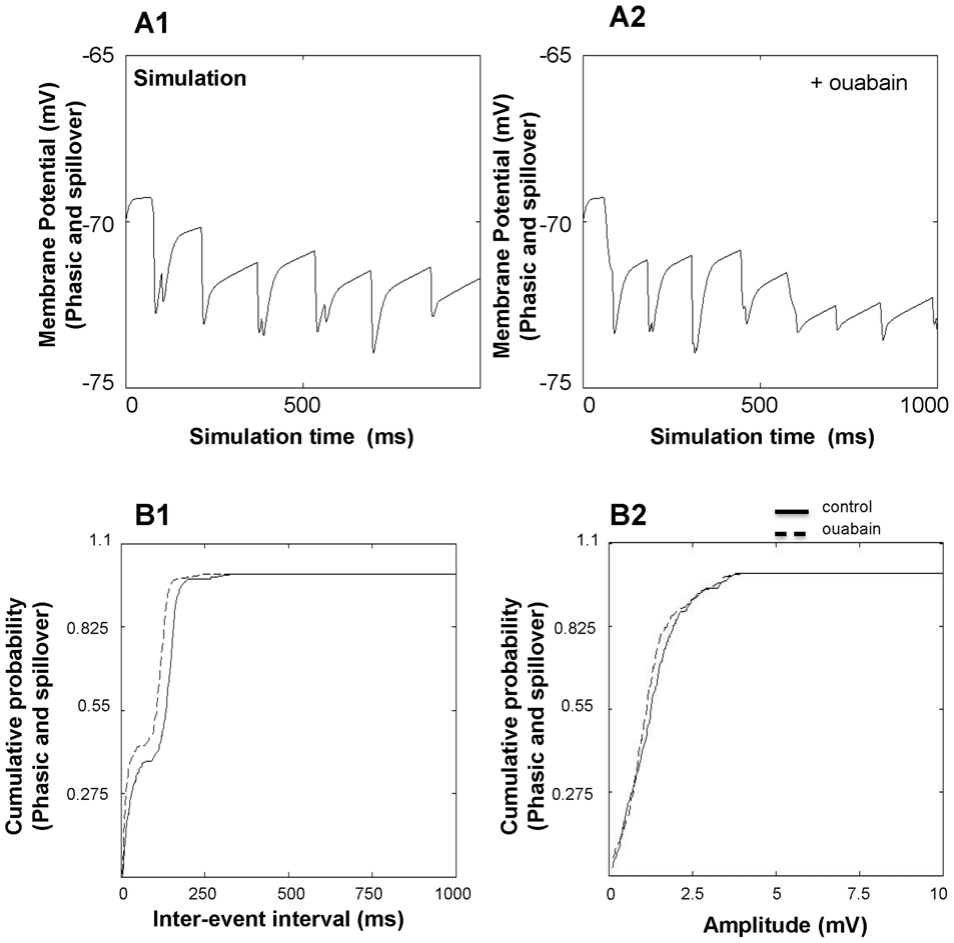
Effect of ouabain on the frequency and amplitude of simulated CGN IPSPs (phasic and perisynaptic components). IPSP traces for (A1) control condition and (A2) in the presence of ouabain including both phasic and perisynaptic “spillover” components. The effect of ouabain on (B1) inter-event interval and (B2) amplitude with both phasic and perisynaptic “spillover” components present. Traces represent recorded inter-event intervals and amplitudes of 50 CGNs.

With just the phasic component present, CGN IPSPs showed a mean inter-event interval of 120.12±5.79 ms for the control condition (Fig. 7A1, B1). Mean amplitude of IPSPs for the same condition was 2.01±0.05 mV (Fig. 7A1, B2). In the presence of ouabain (with just the phasic component present), the inter-event interval of IPSPs decreased to 81.08±2.90 ms (Fig. 7A2, B1; *p*<0.01). The amplitude did not change (Fig. 7B2; 2.01±0.04 mV, *p*>0.05). We then investigated the frequency and amplitude of IPSPs when both the phasic and perisynaptic “spillover” components are present (Fig. 8). Addition of the spillover component decreased the membrane potential by 0.97±0.05 mV. For the control case, the inter-event interval was 101.74±3.43 ms (*p*<0.01 compared to phasic only) and the amplitude of IPSPs was 1.27±0.04 mV (*p*<0.01 compared to phasic only). In the presence of ouabain, the mean inter-event interval decreased to 75.74±2.30 ms (*p*<0.01 compared to no ouabain, *p* = 0.1457 compared to phasic only) with a mean amplitude of 1.18±0.04 mV (*p*>0.05 compared to no ouabain, *p*<0.01 compared to phasic only) (Fig. 8B1, B2; *p*>0.05).

When modeling CGN sIPSPs, we found a decrease in inter-event interval in the presence of ouabain, similar to the experimental results. When simulating phasic transmission only, the decrease in inter-event interval in the presence of ouabain was 32.5±38.7%. Similarly, simulation of phasic and spillover components revealed a ouabain-induced decrease in inter-event interval of 25.5±16.1%. Similar to experimental results, we did not see any significant change in amplitude induced by ouabain.

## Discussion

There has been an ongoing debate as to the source of GABA that mediates tonic currents in the cerebellum. In the current study, we provide evidence further supporting the suggestion that there is a GoC-dependent component of the CGN-I_tonic_ that can be detected even in relatively mature animals. We also found that the Na^+^/K^+^-ATPase inhibitor ouabain can partially mimic the effect of acute EtOH on GABAergic transmission at CGNs. Finally, using computer modeling, we demonstrate that inhibition of the Na^+^/K^+^-ATPase in GoCs results in an increase in IPSPs in CGNs regardless of the presence of a perisynaptic “spillover” component. These findings suggest that the GoC is a common and significant source of GABA for both phasic and tonic inhibitory synaptic transmission within this compact circuitry, and provide additional insight into EtOH's mechanism of action on cerebellar function.

### The GoC-mediated component of CGN-I_tonic_ is not limited to young animals

Previous reports have suggested that GoCs contribute to the CGN-I_tonic_, but only in pre-weaning rodents, as recordings from P35–55 rats showed no GoC-dependent component [8], [9], [10], [14], [15]. However, our current and previous studies argue against this conclusion, as we have consistently found that TTX significantly reduces the CGN-I_tonic_ by ∼30% in 21–45 day old animals [13], [18]. Moreover, Bright et al. also reported a TTX-sensitive CGN-I_tonic_ component in adult mice [7]. One factor that could have contributed to the lack of detection of a GoC-dependent CGN-I_tonic_ component in earlier studies is that it is generally difficult to obtain healthy slices from the brains of mature rodents [29]. It is possible that spontaneous firing of GoCs is compromised in unhealthy slices, leading to a decrease in sIPSCs in CGNs and the GoC-dependent component of CGN-I_tonic_. Consistent with this possibility, CGN-I_tonic_ recordings from 60 day-old mouse slices showed little or no spontaneous phasic currents [11]. Another indicator of compromised slice health could be the presence of large CGN-I_tonic_, as was observed in studies that did not detect a GoC-dependent component (∼42 pA [8], [9], ∼35 pA [11], ∼48 pA [10]) vs. those that did (∼17 pA [13] and ∼16 pA in the current study). Consistent with a potential association between compromised slice health and large tonic conductances, ambient GABA levels were shown to increase in the frontal cortex in a stroke model [30] and in the hippocampus following an ischemic-like insult [31], [32], [33]. Therefore, the current study provides further evidence that in more healthy slices from relatively mature animals there is a component of the CGN-I_tonic_ that is mediated by the GoC.

### Heterogeneity of phasic and tonic current interactions

**Figure 9:**
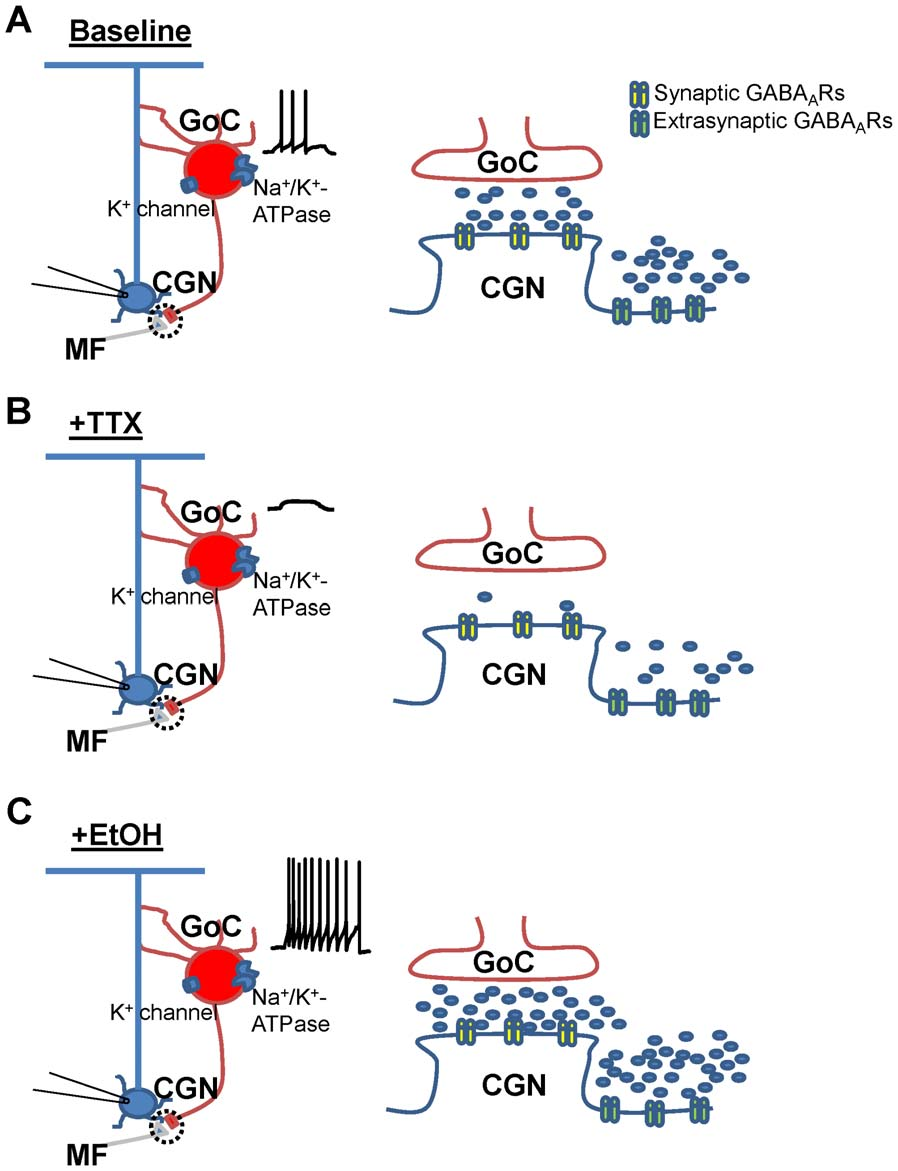
Proposed circuitry of a Golgi cell-granule cell synapse illustrating the effect of TTX and acute EtOH on GABAergic transmission. CGNs make synaptic contacts by both glutamatergic Mossy fibers and GABAergic GoC interneurons. These synapses are surrounded by a glial sheath (black dashed line), forming a glomerulus. (A) Under baseline conditions, GoC firing releases GABA into the synaptic space, mediating phasic GABA_A_R sIPSCs on CGNs (synaptic GABA_A_Rs depicted in yellow). There is also a pool of GABA in the extrasynaptic space mediating CGN-I_tonic_. Spontaneous action potential-dependent GABA release from GoCs contributes to this pool of GABA that activates extrasynaptic GABA_A_Rs (depicted in green) – for clarity purposes, perisynaptic receptors are excluded from the illustration. (B) In the presence of TTX, GoC firing ceases, allowing only quantal release of GABA into the synaptic space and significantly reducing phasic GABA_A_R sIPSCs. Extrasynaptic GABA levels are also reduced, thereby decreasing CGN-I_tonic_. (C) Acute EtOH inhibits the Na^+^/K^+^-ATPase found on GoCs (and possibly also a quinidine-sensitive K^+^-channel [20]). Inhibition of these membrane proteins depolarizes the membrane potential of GoCs, thereby increasing spontaneous GoC firing and phasic GABA release onto CGNs. This leads to a robust increase in the frequency of phasic GABA_A_R-mediated sIPSCs and also increases extrasynaptic GABA levels, resulting in an enhancement of CGN-I_tonic_.

We found no relationship between basal sIPSC frequency and CGN-I_tonic_ and this is consistent with a previous report [7]. This result is expected given that the majority (i.e., ∼70% under our conditions) of the CGN-I_tonic_ is independent of spontaneous action potential firing of GoCs. In contrast to basal conditions, our data show that there is a direct relationship between basal sIPSC frequency and the magnitude of the TTX-induced change on the CGN-I_tonic_. Likewise, when GoC activity is facilitated by acute EtOH, there is a positive correlation between the EtOH-induced change in sIPSC frequency and CGN-I_tonic_. These data suggest that spontaneous action potential-dependent GABA release from GoCs directly contributes to the CGN-I_tonic_, as illustrated in Fig. 9. This further supports the idea that GABA released from GoCs contributes to the GABA pool that activates extrasynaptic GABA_A_Rs in the cerebellar glomerulus. Our findings are in general agreement with those of Glykys et al. [34] who demonstrated a strong correlation between changes in phasic and tonic inhibition in hippocampal neurons under conditions of increased or decreased vesicular GABA release. These findings are important given that synapses in hippocampal neurons lack a glomerulus, which has been suggested to play a role in trapping phasically-released GABA from GoCs, allowing it to contribute to the ambient pool of GABA that activates extrasynaptic receptors [35], [36], [37]. Studies have shown that only ∼40% of CGNs make synaptic contacts with GoCs [38], with more recent studies suggesting this percentage to be even lower (20–26%) [39], [40]. Under our recording conditions, we detected sIPSCs in all CGNs, although in some recordings, these had a very low frequency (Fig. 1). Therefore, these findings suggest that GABA released at GoC-CGN synapses diffuses to neighboring synapses, activating GABA_A_Rs and eliciting sIPSCs via a parasynaptic mechanism [41]. In support of this, paired recordings showed clear examples of a lack of correlation between a GoC spontaneous action potential and a CGN sIPSC [39].

### Ouabain partially mimics the EtOH-induced potentiation of tonic and phasic GABAergic currents

The Na^+^/K^+^-ATPase contributes to the regulation and maintenance of the Na^+^ and K^+^ gradients that are essential for maintaining the resting membrane potential and excitable properties of neurons [42], [43], [44], [45]. Recent studies have demonstrated that an endogenous activator of the Na^+^/K^+^-ATPase, follistatin-like 1, diminishes the excitability of cells [46], while the high affinity Na^+^/K^+^-ATPase inhibitor, ouabain, can depolarize the membrane potential of interneurons in layer 5 of the cortex [47] and increase IPSPs in the hippocampus [48]. Similarly, in the cerebellum, ouabain was shown to depolarize the membrane potential of GoCs [19]. Consistent with this finding, we now show that ouabain potentiates CGN-I_tonic_ amplitude and sIPSC frequency. Furthermore, we also found that, in the presence of TTX, ouabain did not significantly alter either phasic or tonic GABAergic transmission, suggesting that its mechanism of action involves an increase in GoC excitability. The effect of ouabain on CGN-I_tonic_ amplitude was similar to that of EtOH (Fig. 3 and [18], [19], [20]). However, the ouabain-induced increase of sIPSC frequency was not as robust as that of EtOH. In addition, ouabain's effect on sIPSC frequency was not significantly correlated with its effect on CGN-I_tonic_. One possible reason for this is that ouabain is expected to inhibit the Na^+^/K^+^-ATPase in other cell types, such as in glia, which have been suggested to contribute to the CGN-I_tonic_ via the Ca^2+^-activated anion channel, bestrophin 1 [11], [12]. Interestingly, ouabain can increase intracellular Ca^2+^ concentrations in cerebellar astrocytes by altering the Na^+^/Ca^2+^ exchanger [49], and this could potentially result in an increase in the CGN-I_tonic_. However, the lack of an effect of ouabain in the presence of TTX argues against this possibility. Bestrophin 1 channel permeability was shown to be insensitive to acute EtOH, further suggesting that this is not a likely mechanism of EtOH action [13]. Nevertheless, these data suggest that EtOH increases GoC excitability via mechanisms other than inhibition of the Na^+^/K^+^-ATPase. Botta et al. [20] recently showed that the effect of EtOH on GABAergic transmission at CGNs is blocked by quinidine, suggesting that K^+^ channels sensitive to this blocker are an additional target of EtOH in GoCs, potentially explaining the differences between its effect and that of ouabain (Fig. 9).

### Computer modeling of EtOH-mediated facilitation of GABAergic transmission at GoC-CGN synapses

Computer modeling was previously used to reproduce the electrophysiological characteristics of GoCs during inhibition of the Na^+^/K^+^-ATPase by ouabain and EtOH [19]. We have now employed similar computer modeling techniques using previous ouabain modeling parameters, focusing on the phasic activation of synaptic and perisynaptic receptors on CGNs as a result of depolarization-induced GoC firing. The model closely replicated the decrease in inter-event interval of CGN sIPSCs observed in the experimental results in the presence of ouabain (32.5±38.7% for phasic only simulation, 25.5±16.1% for phasic and spillover, and 30.28±17.9% for experimental). These modeling data are also in agreement with the Na^+^/K^+^-ATPase being only one of the targets for EtOH since this ouabain-induced decrease in inter-event interval is less than what this study found using 80 mM EtOH (150.1±43.62%) or by previous reports using 75 mM EtOH (110%) to sIPSCs [18]. Similar to experimental results, we did not see any significant change in amplitude induced by ouabain in the presence or absence of the spillover component.

### Conclusions

The current study provides evidence suggesting that GABA release driven by spontaneous firing of GoCs contributes to the pool of ambient GABA that generates CGN-I_tonic_. We provide further evidence that Na^+^/K^+^-ATPase inhibition is one of the mechanisms responsible for the acute EtOH-induced increase of GABAergic transmission in CGNs. Given that GoCs are connected through gap junctions [50], and more recently have been shown to be synaptically inhibited by reciprocal connections [40], the effects of EtOH on these neurons are likely to have a significant impact on the processing of incoming information into the cerebellum, and to be one of the underlying mechanisms of the de-afferentation of the cerebellar cortex from mossy fiber afferents that has been associated with acute EtOH intoxication [21].
